# Degradation of crude oil in a co-culture system of *Bacillus subtilis* and *Pseudomonas aeruginosa*

**DOI:** 10.3389/fmicb.2023.1132831

**Published:** 2023-05-12

**Authors:** Bo Wu, Jianlong Xiu, Li Yu, Lixin Huang, Lina Yi, Yuandong Ma

**Affiliations:** ^1^School of Engineering Science, University of Chinese Academy of Sciences, Beijing, China; ^2^Institute of Porous Flow and Fluid Mechanics, University of Chinese Academy of Sciences, Beijing, China; ^3^State Key Laboratory of Enhanced Oil Recovery, PetroChina Research Institute of Petroleum Exploration and Development, Beijing, China; ^4^PetroChina Research Institute of Petroleum Exploration and Development, Beijing, China

**Keywords:** co-culture, biosurfactant, biodegradation, *Bacillus subtilis*, *Pseudomonas aeruginosa*

## Abstract

Microbial remediation has been regarded as one of the most promising decontamination techniques for crude oil pollution. However, there are few studies on the interaction of bacteria in the microbial community during bioremediation. The aim of this work was to research the promotion of defined co-culture of *Bacillus subtilis* SL and *Pseudomonas aeruginosa* WJ-1 for biodegradation of crude oil. After 7 days of incubation, the analysis of residual oil, saturated and aromatic fraction in the samples showed that the degradation efficiency of them was significantly improved. The degradation efficiency of crude oil was enhanced from 32.61% and 54.35% in individual culture to 63.05% by the defined co-culture of strains SL and WJ-1. Furthermore, it was found that the defined co-culture system represented relatively excellent performance in bacterial growth, cell surface hydrophobicity (CSH) and emulsification activity. These results indicated that the combination of *Bacillus subtilis* and *Pseudomonas aeruginosa* can effectively promote the degradation and utilization of crude oil, which may provide a new idea for the improvement of bioremediation strategies.

**GRAPHICAL ABSTRACT:**
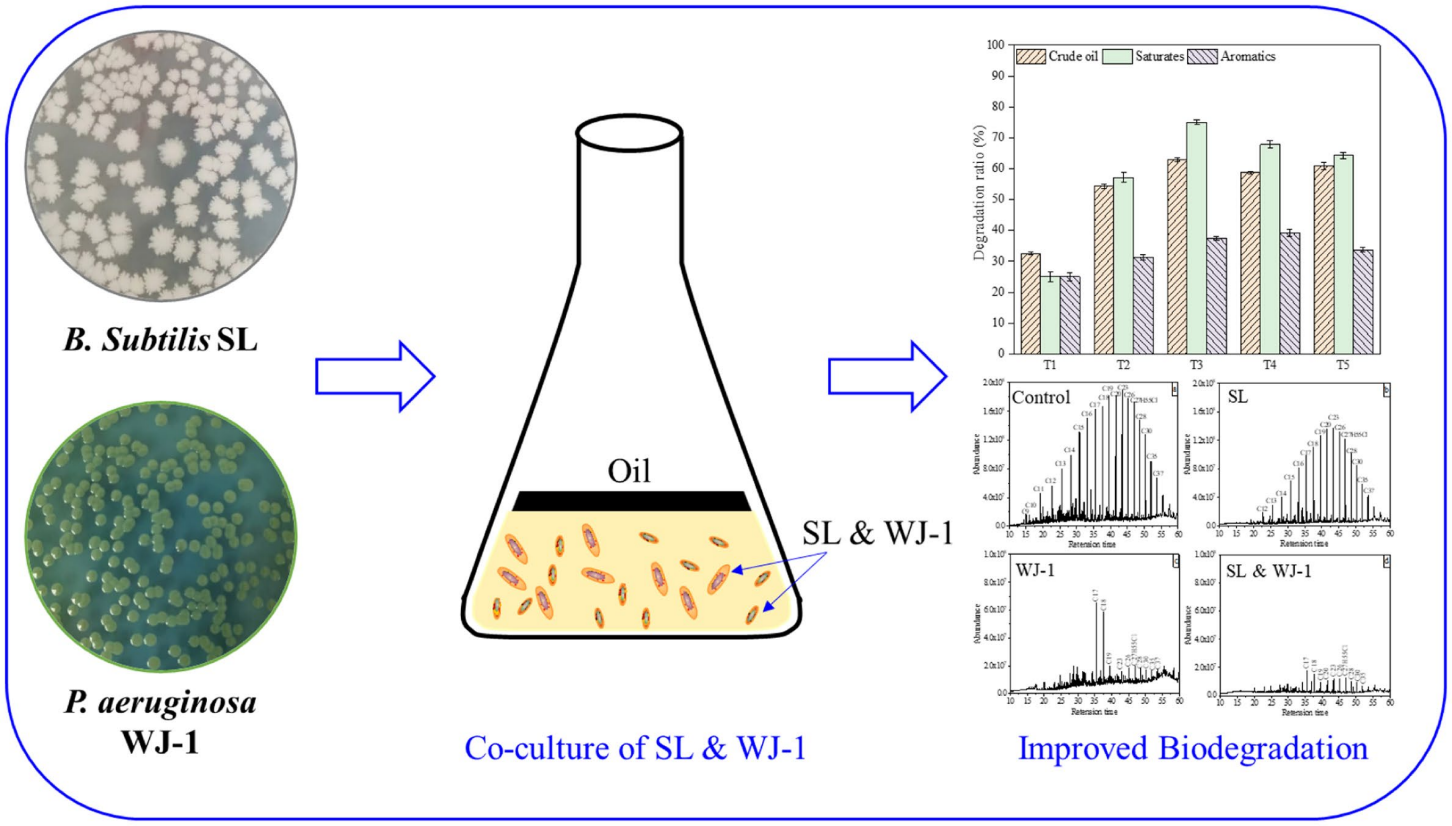

## Highlights

- Crude oil degradation was enhanced by co-culture of *Bacillus subtilis* and *Pseudomonas aeruginosa*.
- About 75% saturated fractions of crude oil were effectively removed by the defined co-cultures.
- The cell density and cell surface hydrophobicity of microbial community were enhanced by co-cultures.

## 1. Introduction

Petroleum is an important material basis for human survival and development. However, since the onset of petroleum exploration and utilization, the environmental contamination by petroleum and its derivatives has always been a global concern and was once considered the most common environmental pollutants (Tao et al., 2020; Hentati et al., 2021). During oil exploration, transportation and utilization, the contamination of soil and waterbodies by the accidental leakage of crude oil has caused extremely bad influence on the ecosystem (Zargar et al., 2022). Furthermore, crude oil contains many different types of chemical components of which are toxic, and long-term exposure to these substances can seriously damage human health (Varjani, 2017). Therefore, an effective remedial measure is urgently needed to repair the polluted environment. The treatment of crude-oil-contaminated environments can be divided into physical remediation, chemical remediation and biological remediation (Parthipan et al., 2017). Among them, the operation cost of physical and chemical remediation is high, and it is easy to cause secondary pollution (Muthukumar et al., 2022). Bioremediation is viewed as one of the most promising environmental remediation methods on account of its low cost and ability to transform pollutants into non-toxic and harmless final products (Tao et al., 2020; Goveas et al., 2022; Zargar et al., 2022).

Although an abundance of petroleum hydrocarbon degrading microorganisms have been discovered in nature, two principal elements limit their application in bioremediation. First, petroleum is a mixture of many simple and complex hydrocarbons, and a single type of microorganism can degrade only a limited range of hydrocarbons. Secondly, the low water solubility of petroleum hydrocarbons limits the utilization and degradation of microorganisms to a certain extent. To enhance the efficiency of microbial bioremediation, it is necessary to expand the biodegradation range of petroleum hydrocarbons and overcome their mass transfer resistance in aqueous solutions.

The co-culture of multiple microorganisms is a common method to expand the biodegradation of petroleum hydrocarbons, which is considered as a promising treatment technology for petroleum contaminated ecosystem. Studies have shown that under co-culture conditions, the performance of microbial consortium shows better than individual microorganisms due to the collaboration between different microorganisms, such as co-metabolism or antagonism (Li et al., 2020; Park et al., 2021; Yun et al., 2021). The scientists co-cultured *Sarocladium* sp. and *Cryptococcus* sp., and found the biodegradation efficiency was a 28% higher than that of individual cultures (Kamyabi et al., 2017). Similar studies, such as the co-culture of *Rhodococcus* sp. WB9 and *Mycobacterium* sp. WY10, and the co-culture of *Escherichia coli* HY1 *and P. aeruginosa* PH2, showed better than individual cultures in the biodegradability of petroleum hydrocarbon (Jia et al., 2019; Sun et al., 2021).

As to how to reduce the mass transfer resistance, the use of surfactant is considered to be a feasible method. Surfactants as amphiphilic molecules, through the role of hydrophobic groups and hydrophilic groups, reduce the oil–water interfacial tension, improve the solubility or mobility of crude oil in the aqueous phase, which helps to increase the emulsification of hydrophobic pollutants, put on the contact area between petroleum hydrocarbons and bacteria, improve the bioavailability of petroleum hydrocarbons, so as to promote biodegradation. Several studies have demonstrated the effectiveness and performance of surfactant assisted bioremediation. Glycolipid biosurfactants supplementation resulted in up to 23.5% improvement in degradation of C10–C24 alkanes by *P. putida* (Sajna et al., 2015). It was found that biosurfactants produced by *Acinetobacter calcoaceticus* BU03 can increase the aqueous solubility of phenanthrene and pyrene, even higher than the synthetic surfactants Tween-80, Brij-58, and Triton X-100 (Zhao et al., 2011; Zhou et al., 2011).

*Bacillus subtilis* and *Pseudomonas aeruginosa*, as the petroleum-degrading bacteria and biosurfactant-producing bacteria, have tremendous application potential in bioremediation. Studies showed that the saturates and aromatics in crude oil can be degraded by *Pseudomonas* and *Bacillus*, especially *Pseudomonas* can degrade resin and asphaltene as well. Furthermore, biosurfactants produced by *Pseudomonas* and *Bacillus* also have good emulsification and solubilization on crude oil. However, there are few studies on the co-culture of these two excellent strains and their interaction to remove pollutants.

The primary purpose of this work was to research the bioremediation potential of petroleum contaminants by a defined co-culture of *Bacillus subtilis* SL and *Pseudomonas aeruginosa* WJ-1. The optimum incubation ratio of co-culture strains and the role of different microorganisms in the process of crude oil degradation were discussed. Moreover, the differences of individual cultures and co-cultures of the two strains in the production of biosurfactant, emulsification activity, cell surface hydrophobicity (CSH) and crude oil biodegradation were conducted as well. On present understanding, research on the relationship between these factors has not been carried out detailed, and this work will contribute to comprehending the synergistic effect of *Pseudomonas* and *Bacillus* co-culture on crude oil degradation.

## 2. Materials and methods

### 2.1. Chemical and culture medium

The crude oil (density: 0.806 g/cm^3^) used in this study was obtained from Changqing Oilfield, China. All other chemicals, reagents and hydrocarbons, analytical grade or better, were purchased from Sinopharm Chemical Reagent Co., Ltd., China. The Luria-Bertani (LB) medium contained yeast extract (5.0 g/l), NaCl (10.0 g/L), peptone (10.0 g/L). Solid medium, agar (20.0 g/L) was added into the solution before autoclaving. The degradation experimental medium with crude oil as the sole carbon source contained NaNO_3_ (3.0 g/L), K_2_HPO_4_·H_2_O (4.0 g/L), KH_2_PO_4_ (3.4 g/L), MgSO_4_·7H_2_O (0.6 g/L). The pH was adjusted to 7.0 using HCl (1 mol/L) and NaOH (1 mol/L) before sterilization at 121°C for 20 min.

### 2.2. Microbial culture and inoculum preparation

The strain WJ-1 used in this study was *Pseudomonas aeruginosa*, isolated previously from the brine and oil samples in our laboratory (Xia et al., 2012). It was identified by 16S rDNA gene sequencing method and submitted to NCBI GenBank database under the accession numbers FJ948174. The strain SL was isolated from oil and water samples of Daqing Oilfield. The results of 16S rDNA sequence analysis showed that strain SL with 99% sequence similarity with *Bacillus subtilis* B7 (KC310823.1), was confirmed to be *Bacillus subtilis* (Wu et al., 2022). Both the organisms were maintained in 30%, v/v sterile glycerol solute ion at −80°C.

The two strains were grown in LB liquid medium at 180 rpm and 37°C until the exponential phase was reached. By this time, the concentration of strains WJ-1 and SL were about 2.1 × 10^11^ ± 0.23 × 10^11^ and 1.0 × 10^10^ ± 0.23 × 10^10^ colony-forming units per milliliter (CFUs mL^−1^). Thereafter, the absorbance (OD_600_) of seed culture was adjusted to 1.0 with sterile water by using a AQ7100 spectrophotometer (Thermo Fisher Scientific, United States), and then placed in 4°C environment for reserve.

### 2.3. Crude oil removal test by *Bacillus subtilis* SL and *Pseudomonas aeruginosa* WJ-1

In order to research the degradation of crude oil by the experimental strains, six treatments were carried out in 250 mL Erlenmeyer flasks, which containing 100 mL degradation culture medium. The six experimental treatments were designed as follows: crude oil degradation medium without inoculum as negative control (T0); crude oil degradation medium inoculated with *Bacillus subtilis* SL (T1); degradation medium inoculated with *Pseudomonas aeruginosa* WJ-1 (T2); degradation medium inoculated with WJ-1 and SL at a ratio of 1:1 for co-culture (T3); degradation medium inoculated with WJ-1 and SL at a ratio of 1:2 for co-culture (T4); degradation medium inoculated with WJ-1 and SL at a ratio of 2:1 for co-culture (T5). All the flasks supplemented with inoculum (3% v/v) were cultured 7 days in an orbital shaker incubator at 37°C and 180 rpm.

After biodegradation, the culture medium was added to a 500 mL separatory funnel with 100 mL petroleum ether to extract residual oil (60–90°C), and repeated 2–3 times (Tao et al., 2020). The dissolved phase was then collected and dried at room temperature before weighing. Compared with the control group, the biodegradation efficiency of crude oil in each sample was determined. Saturated hydrocarbons, aromatic hydrocarbons, resins and asphaltenes were separated from the residual oil samples with test method for separation of asphalt into four fractions (SH/T0509-2010) according to Petrochemical Industry Standard of the People’s Republic of China (He et al., 2016). Then the hydrocarbons of residual oil were analyzed by a gas chromatography combined with mass spectrometry instrument (GC–MS; Agilent, United States), which is equipped with an HP-5AS capillary column (30 m × 0.25 mm × 0.25 μm, Agilent, United States). The execution program of GC–MS is as follows: 40°C for 10 min, raised to 295°C at the speed of 5°C/min, held at 295°C for 30 min. Helium was used as the carrier gas, the column flow rate was 1 μL/min, the injector temperature was 295°C and the split ratio was 29:1. The ion source was obtained by electron ionization mode at 70 eV. The temperature of ion source and four-stage bar was 230 and 150°C, respectively. The scanning range was from m/z 40 to 550.

### 2.4. Determination of cell density and surface tension

After biodegradation, 50 mL of the culture medium was centrifuged at 1,000 rpm and 4°C for 10 min, rinsed three times with sterile water, and then diluted to the original volume with sterile water. The density of cells in the culture medium was determined by detecting the optical density at 600 nm wavelength with ultraviolet spectrophotometer. It is worth noting that the optical density measured by the spectrophotometer is an indirect reflection of the concentration of cells in the suspension. When visible light passes through the cell suspension, the light is scattered. The higher the scattering intensity, the higher the bacterial concentration in the suspension. Therefore, the centrifuged bacteria were washed three times, to reduce the influence of other suspended solids in the culture medium. The cell-free supernatant was collected after the initial centrifugation and its surface tension was measured with a surface tensiometer FTA1000B (First Ten Angstroms, Portsmouth, Virginia, United States) to indirectly check whether biosurfactants were produced during biodegradation. Beforehand, the surface tension of distilled water was measured for instrument calibration.

### 2.5. Measurement of emulsifying activity

The emulsification activity of biosurfactants produced by microbial metabolism on different hydrocarbons such as crude oil, hexane, hexane and diesel oil was estimated by emulsification index. The cell-free supernatant was mixed with hydrocarbons described above with equal volume (2 mL) in a test tube (Joy et al., 2017). Then the samples were vortexed for 2 min and allowed to stand for 24 h at room temperature. The emulsification index was calculated from the following equation.

$$\begin{array}{c} \mathrm{Emulsification}\\ \mathrm{Index}\left({\mathrm{EI}}_{24}\right) \end{array}=\frac{\mathrm{Height of the emulsion layer}}{\mathrm{Height of organic phase layer}\;\;}\times 100\%$$

### 2.6. Measurement of cell surface hydrophobicity

The CSH of bacteria was determined by bacterial adhesion to hydrocarbons (BATH) method (Pandey et al., 2022). After biodegradation, the cell broth was centrifuged at 6,000 rpm for 15 min at 4°C, then washed with sterile water, and the process was repeated twice. Then the optical density (OD) of the suspension was adjusted to 0.3 ~ 0.4 at 600 nm. The 5 mL cell suspension was added with 0.5 mL of n-hexadecane, and the control group was not added with organic phase and sealed with rubber stopper. Swirl for 2 min at room temperature, then pour into a Teflon burette and let stand for 30 min until the two phases (aqueous and hydrophobic) separate. Then the OD value of the aqueous phase was determined. The affinity of bacterial cells for hydrocarbons was calculated by the following equation, and the high hydrophobicity, medium hydrophobicity, and poor hydrophobicity of bacteria were distinguished by CSH exceeding 50%, between 20% and 50%, and below 20%.

$$\mathrm{CSH}\%=\frac{\begin{array}{l} {\mathrm{microbial concentration}}_{\mathrm{control}}\\ -{\mathrm{microbial concentration}}_{\mathrm{treatment}\;\;} \end{array}}{{\mathrm{microbial concentration}}_{\mathrm{control}}}\times 100\%$$

where microbial concentration _control_ is the optical density of the initial cell suspension at 600 nm and microbial concentration _treatment_ is the optical density of the cell suspension at 600 nm after treatment with n-hexane.

### 2.7. Statistical analysis

All the experiments were replicated three times and experimental data were means ± standard deviation (*n* = 3).

## 3. Results and discussion

### 3.1. Study of bacterial growth

**Figure 1:**
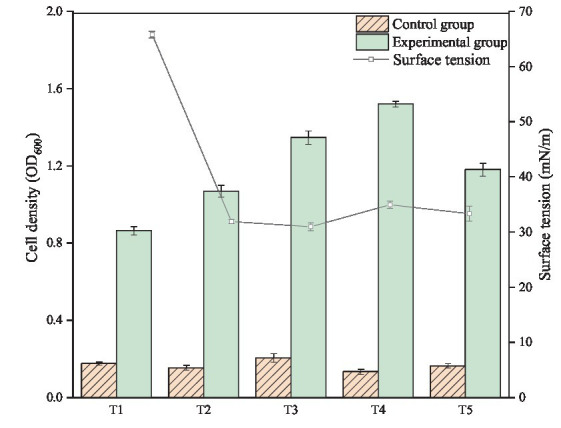
Cell growth and surface tension changes of individual and co-culture of *Bacillus subtilis* and *Pseudomonas aeruginosa* after 7 days of incubation.

The target microorganisms exhibit ability to degrade crude oil pollutants to maintain their growth is the key in bioremediation process. In the experiment, the cell density of each sample was measured after 7 days of culture to observe the growth of bacteria. As shown in Figure 1, without carbon sources, the OD600 of control groups were maintained at approximately 0.2 during the monitoring period, indicating that all the test bacteria strains cannot grow in inorganic medium lacking carbon source. However, the OD value and cell density of the corresponding experimental group were significantly increased. Thus, all the experimental strains could metabolize and proliferate in the medium with crude oil as the sole carbon source. The growth of bacteria in T4 group was the best, and its OD value reached 1.520. The OD600 values of T3 and T5 also reached 1.346 and 1.180, respectively. The OD600 values of T1 and T2 were lower, which were 0.864 and 1.068, respectively.

The cell density of co-culture group was significantly higher than that of individual culture group. The results showed that *Bacillus subtilis* and *Pseudomonas aeruginosa* had some synergies in the degradation and utilization of crude oil. However, little is known about the interaction between the two strains of bacteria in crude oil degradation. Therefore, it is necessary to analyze the crude oil before and after biodegradation and further explore the effect of bacterial co-culture on the components of the crude oil.

### 3.2. Evaluation of surface tension and emulsification activity

Considering the ability of *Bacillus subtilis* and *Pseudomonas aeruginosa* to produce biosurfactants, the production of biosurfactants in crude oil biodegradation process was monitored indirectly by measuring the surface tension of the six groups (T0, T1, T2, T3, T4, and T5). Previous studies (as shown in Table 1) have shown that the surface tension of *Pseudomonas aeruginosa* WJ-1 broth can be reduced to 24.50 mN/m with vegetable oil as the sole carbon source, and the surface tension of *Bacillus subtilis* SL broth can decline to 25.65 mN/m while using sucrose as sole carbon source substrate (Xia et al., 2012; Wu et al., 2022). Thus, it can be seen that the two strains have an outstanding ability to decline surface or interfacial tension. However, surprisingly, there was no significant difference in the surface tension between the T0 and T1 groups in biodegradation test, which indicating that *Bacillus subtilis* SL almost not produced surface active substance in the conditions. In all instances, biosurfactant production is positively correlated with cell density (Ron and Rosenberg, 2002). As shown in Figure 1, the growth of *Bacillus subtilis* SL was not significantly inhibited, and the production of biosurfactant was not detected in the experiment. This indicates that the reproductive capability of strain SL has not concerned with its metabolic capacity of biosurfactant.

**Table 1:** Comparison of surface tension in various samples with different carbon sources.

Samples	Carbon sources	Surface tension (mN/m)	References
Culture medium (T0)	Crude oil	66.95 ± 0.75	This study
*Bacillus subtilis* SL	Sucrose	25.65 ± 0.64	Wu et al. (2022)
*Pseudomonas aeruginosa* WJ-1	Waste vegetable oils	24.50	Xia et al. (2012)
SDS	/	34.00 ± 0.50	Preethy and Nilanjana (2010)
DTAB	/	36.20	Liu et al. (2022)
Tween 80	/	37.18	Meshram et al. (2021)
Saponin	/	37.39	Meshram et al. (2021)

As shown in Figure 1 and Table 1, in this study, the surface tensions of all the culture media (except T0 and T1) were below 35 mN/m. In comparison with the biosurfactants produced by experimental strains, the chemical surfactants such as Sodium dodecyl sulfate (SDS), Cetyltrimethylammonium bromide (DTAB) and Tween 80 are slightly inadequate in reducing surface tension. When crude oil was used as carbon source, the highest surfactant production was achieved in the condition of co-culture (T3). Compared with SDS and Tween 80, it could further reduce the surface tension by 9.82% and 20.09%, respectively. Moreover, by comparing the surface tension of microbial fermentation broth with different carbon sources, it is not hard to see that when crude oil as the only carbon source, the performance of individual cultures in producing surfactant behave badly. Even in co-culture conditions, biosurfactant production is also quite limited during the biodegradation of crude oil. In fact, the carbon source is an extremely important factor in the production of biosurfactants by microorganisms. Carbohydrate is considered as an ideal carbon source, and petroleum has previously been reported to inhibit biosurfactant production (Tao et al., 2017).

**Figure 2:**
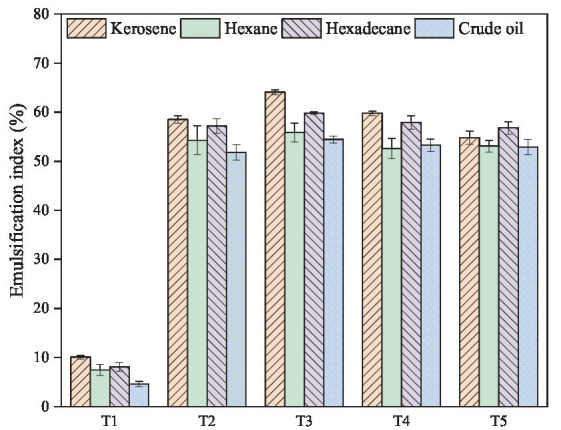
Emulsification activity against different hydrocarbons of the cell-free fermentation broth of individual and co-culture of *Bacillus subtilis* SL and *Pseudomonas aeruginosa* WJ-1 containing the crude biosurfactant.

Furthermore, the surfactant produced by microorganisms is usually specific and has different emulsifying abilities for different hydrocarbons. When it interacts with hydrocarbons, it can form an emulsion and increase the contact area between bacteria and hydrocarbons, thus improving the biodegradation efficiency. In this study, emulsification index values were used to evaluate the emulsification activity of the microbial fermentation broth with four different hydrocarbons, including hexane, kerosene, hexadecane and crude oil. It was found that T2-T5 groups all exhibited significant emulsification activity against different hydrocarbons, and the co-culture group (T3) had the best whole effect (Figure 2). The highest emulsification index value of T3 group was observed for kerosene (64.07%), followed by those obtained for hexadecane (59.81%), hexane (55.86%), and crude oil (54.42%). A comparison between individual culture and co-culture, the co-culture group (T3) showed further improved emulsification activity of different hydrocarbons, by 9.54%, 4.60%, 2.94%, and 5.08% than individual culture group T2. These results indicated that there was a synergistic effect between *Bacillus subtilis* and *Pseudomonas aeruginosa* in mixed culture (Table 2).

**Table 2:** Comparison of cell surface hydrophobicity in various samples with different culture media.

Samples	LB medium/%	Degradation medium/%
*Bacillus subtilis* SL	16.06 ± 0.38	23.10 ± 0.92
*Pseudomonas aeruginosa* WJ-1	21.04 ± 0.79	46.80 ± 0.59
SL and WJ-1	37.14 ± 0.98	52.46 ± 1.17

### 3.3. Variation of cell surface hydrophobicity

Most petroleum hydrocarbons are insoluble in water, and microbial degradation of these substances requires increased exposure to them. Secretion of extracellular biosurfactant to improve the accessibility of hydrocarbons and change the hydrophobicity of cell surface to enhance the interaction between hydrocarbons and cells are two key strategies for microbial degradation of hydrophobic hydrocarbons (Chen et al., 2020; Carolin et al., 2021). CSH is an important response of microorganisms to environmental factors, which can affect the adhesion and degradation of organic compounds (Yuan et al., 2018). High CSH facilitates microbial adhesion to the hydrocarbon-hydrocarbon-interface, thereby facilitating the direct absorption and utilization of hydrocarbons at the interface (Zhou et al., 2018). Therefore, CSH has become an important parameter to evaluate the adhesion ability of microorganisms to hydrocarbon matrix.

In present study, the CSH of co-culture bacteria was 52.46% against hexadecane, which was 127.10% and 12.09% higher than individual culture of *Bacillus subtilis* SL and *Pseudomonas aeruginosa* WJ-1, respectively. Moreover, a comparison of CSH in various samples with different culture mediums, it was found that the surface hydrophobicity of bacteria in crude oil medium was significantly enhanced. Similar studies have shown that *Candida* grown on hydrophobic compounds produced stronger surfactants and surface hydrophobicity than those grown on glucose medium (Coimbra et al., 2009). Analysis of the reason may be environmental changes, including changes in nutrients and competition among bacteria, which lead to the change of surface properties of bacteria by regulating their own surface proteins, so as to achieve their own growth, reproduction and diffusion.

### 3.4. Crude oil biodegradation by co-culture of *Bacillus subtilis* SL and *Pseudomonas aeruginosa* WJ-1

**Figure 3:**
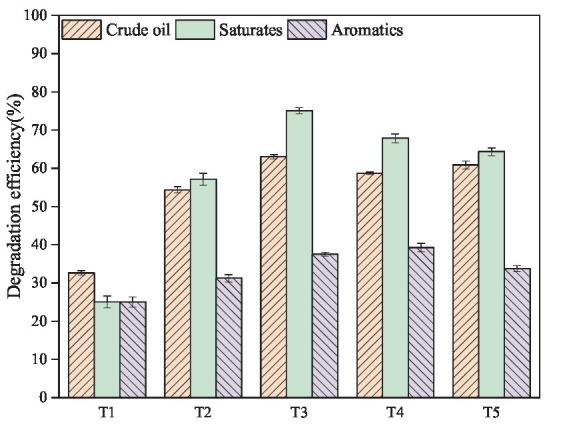
Degradation efficiency of crude-oil, saturates and aromatics fraction after 7 days of incubation.

The ability of microorganisms to degrade crude oil allows them to grow on crude oil as their sole carbon source and to produce a variety of biological products. In the biodegradation experiment, the composition and morphology of crude oil can be clearly observed to change. Crude oil begins as a thin film floating on a water-based medium. Later, under the action of microorganisms, the crude oil is transformed into small droplets, which are dispersed in the aqueous phase. Finally, the droplets are completely homogenized and mixed into the medium. After the biodegradation experiment, the content changes of crude oil and its components before and after microbial degradation were analyzed by weighing method and thin layer chromatography to determine the degradation efficiency of different experimental groups (Wang et al., 2020, 2022). In terms of crude oil removal, as shown in Figure 3, individual cultures and co-cultures all exhibited excellent biodegradation ability, with a removal efficiency of 32.61% for *Bacillus subtilis* SL (T1), 54.35% for *Pseudomonas* sp. WJ-1 (T2), and a significant increase in removal efficiency over 58.60% for SL and WJ-1 (T3, T4, T5). The degradation of saturates, aromatics, resins, and asphaltenes in crude oil was also studied (full details are given in Supplementary Table S1). It is found that the degradation efficiency of saturated hydrocarbon components is more obvious than other components. The main reason is that the saturated hydrocarbon is more easily decomposed and used by bacteria. Among them, the T3 group has the best degradation ability of saturated hydrocarbon, reaching 75.06%. However, there was no significant difference in the degradation ability of aromatic hydrocarbons and other components among all groups, which were less than 40%.

**Figure 4:**
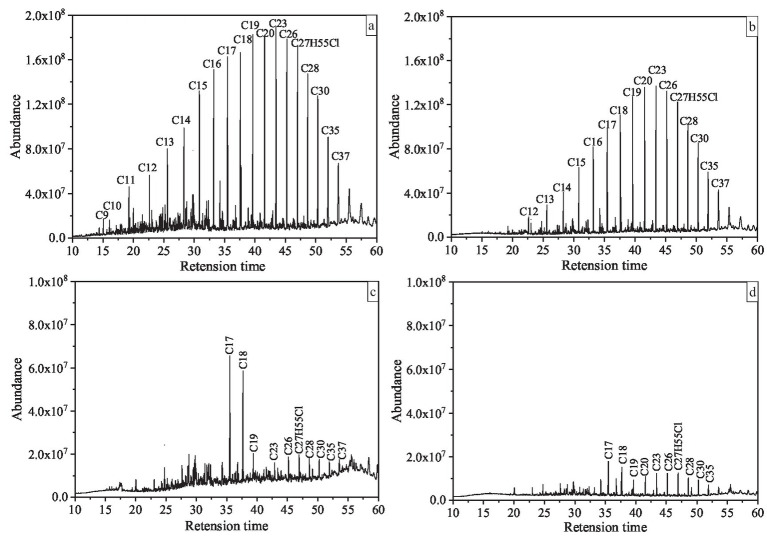
Gas chromatography–mass spectrometry chromatographs of crude oil degraded by co-culture of *Bacillus subtilis* SL and *Pseudomonas aeruginosa* WJ-1. **(A)** Abiotic control. **(B)** Crude oil degradation with SL. **(C)** Crude oil degradation with WJ-1. **(D)** Crude oil degradation with SL and WJ-1. All chromatographs are of the same intensity.

In addition, in order to further study the degradation of saturated hydrocarbon components (n-alkanes) in T3, we selected T0, T1, T2, T3 for GC–MS analysis (Figure 4). Taking T0 as the control group, it was found that the C9–C37 n-alkanes in T1 were degraded overall, but a large amount of them were still not degraded. The n-alkanes of C9-C16 in T2 group were almost completely degraded, but the n-alkanes of C17–C37 were not completely degraded, and some residues were still present. However, the n-alkanes of C9–C37 in T3 group were almost completely degraded after 7 days of incubation, especially the contents of C17 and C18, which were significantly reduced compared with T2. To some extent, this indicates that the ability of the mixed culture to completely degrade crude oil pollutants is better than that of the pure culture under the synergistic effect of each member.

In addition, the inoculation ratio of each member in the co-culture may also have a significant impact on the degradation effect of crude oil. Therefore, in this study, the mixing ratio of *Bacillus subtilis* and *Pseudomonas aeruginosa* was adjusted to make the mixed culture coordinated so as to promote the degradation of crude oil. Studies have found that the optimal inoculation ratio of *Bacillus subtilis* and *Pseudomonas aeruginosa* was SL/WJ-1 = 1:1. The co-cultures at this ratio performed excellent in degrading crude oil and saturated hydrocarbon, with a removal efficiency of 63.05% and 75.06%, respectively. Even compared with previous studies such as *Sarocladium* sp. and *Cryptococcus* sp. (50%), *Pseudomonas aeruginosa* and *Rhodococcus* sp. (40%), *Scedosporium* sp. and *Acinetobacter* sp. (58.61%), the defined co-culture of *Bacillus subtilis* and *Pseudomonas aeruginosa* showed excellent performance in degrading crude oil as well (Hamme and Ward, 2001; Kamyabi et al., 2017; Atakpa et al., 2022).

## 4. Conclusion

The aim of this study was conducted to investigate the combined effect of biosurfactant-producing bacteria, *Pseudomonas aeruginosa* WJ-1 and *Bacillus subtilis* SL on crude oil degradation. Compared with isolated cultures of WJ-1 and SL, co-culture showed enormous potential in bacterial growth, CSH, emulsification and degradation of crude oil. Especially when the inoculation ratio of *Pseudomonas aeruginosa* and *Bacillus subtilis* was 1:1, the degradation efficiency of crude oil reached 63.05%, and the n-alkanes were almost completely degraded. This work demonstrates the application potential of defined co-cultures in bioremediation. However, on the whole, the mechanism of biodegradation of crude oil by co-cultures is indistinct. Therefore, further study should aim at the action and philosophy of bacterial co-culture technology in bioremediation of oil-contaminated soil in order to construct microbial communities with excellent remediation performance.

## Data availability statement

The original contributions presented in the study are included in the article/Supplementary material, material, further inquiries can be directed to the corresponding author.

## Author contributions

BW: conceptualization, methodology, investigation, and writing-original draft. JX: funding acquisition, project administration, resources, and writing review and editing. LYu: funding acquisition, investigation, writing-reviewing and editing, and project administration. LH: funding acquisition and writing-reviewing and editing. LYi: formal analysis and project administration. YM: writing review and editing and formal analysis. All authors contributed to the article and approved the submitted version.

## Conflict of interest

The authors declare that the research was conducted in the absence of any commercial or financial relationships that could be construed as a potential conflict of interest.

## Publisher’s note

All claims expressed in this article are solely those of the authors and do not necessarily represent those of their affiliated organizations, or those of the publisher, the editors and the reviewers. Any product that may be evaluated in this article, or claim that may be made by its manufacturer, is not guaranteed or endorsed by the publisher.
