# Supplementary material for: Degradation of crude oil in a co-culture system of Bacillus subtilis and Pseudomonas aeruginosa
Source: Front Microbiol. 2023 May 12;14:1132831. doi: 10.3389/fmicb.2023.1132831 (PMC10213283; doi:10.3389/fmicb.2023.1132831)
Supplement: Supplementary file 1 [file Table_1.docx]

**Supplementary file for**

**Degradation of crude oil in a co-culture system of Bacillus subtilis and Pseudomonas aeruginosa**

Bo Wu ^1,2,3^, Jianlong Xiu^4^, Li Yu ^4^, Lixin Huang ^4^, Lina Yi ^4^, Yuandong Ma ^4^

1. School of Engineering Science, University of Chinese Academy of Sciences, Beijing, China

2. Institute of Porous Flow and Fluid Mechanics, University of Chinese Academy of Sciences, Beijing, China

3. State Key Laboratory of Enhanced Oil Recovery, PetroChina Research Institute of Petroleum Exploration and Development, Beijing, China

4. PetroChina Research Institute of Petroleum Exploration and Development, Beijing, China

Corresponding author at: PetroChina Research Institute of Petroleum Exploration and Development, China.

E-mail addresses: xiujianlong69@petrochina.com.cn (J. Xiu).

Table S1 The four fractions of crude oil before and after bacterial treatment

| Sample | SARA composition | | | |
| --- | --- | --- | --- | --- |
|  | Saturates/mg | Aromatics/mg | Resins/mg | Asphaltenes/mg |
| T0 | 1789±16 | 1089±13 | 311±12 | 196±9 |
| T1 | 1206±10 | 817±16 | 233±4 | 143±6 |
| T2 | 816.6±13 | 467±17 | 214±3 | 126±3 |
| T3 | 661±10 | 272±9 | 194±2 | 122±5 |
| T4 | 739±6 | 350±13 | 189±3 | 134±2 |
| T5 | 700±18 | 389±11 | 206±3 | 125±8 |
